# Levodopa-responsive dystonia, parkinsonism, and treatment-resistant schizoaffective disorder in Williams syndrome

**DOI:** 10.1007/s10072-024-07705-3

**Published:** 2024-07-18

**Authors:** Nikolai Gil D. Reyes, Nathaniel Bendahan, Emily Swinkin, Anthony E. Lang, Anne S. Bassett

**Affiliations:** 1 Edmond J. Safra Program in Parkinson’s Disease and the Morton and Gloria Shulman Movement Disorders Clinic, Toronto Western Hospital, Toronto, ON Canada; 2 Institute of Medical Science, Temerty Faculty of Medicine, University of Toronto, Toronto, ON Canada; 3 The Dalglish Family 22q Clinic, University Health Network, Toronto, ON Canada; 4 Division of Cardiology, Centre for Mental Health & Toronto General Hospital Research Institute, University Health Network, Toronto, ON Canada; 5 Clinical Genetics Research Program, Centre for Addiction and Mental Health, Toronto, ON Canada; 6 Campbell Family Mental Health Research Institute, Toronto, ON Canada; 7 Department of Psychiatry, University of Toronto, Toronto, ON Canada

**Keywords:** Dystonia, Parkinsonism, Levodopa, Schizoaffective disorder, Williams Syndrome

## Abstract

**Background:**

Williams syndrome (WS; chromosome 7q11.23 deletion) is a rare, multisystemic, neurodevelopmental disorder with variable penetrance and expressivity. Although movement and psychiatric disorders are known to occur in individuals with WS, parkinsonism, dystonia, and treatment-resistant schizoaffective disorder have not been formally described.

**Methods:**

We present two unrelated cases of adults with molecularly confirmed WS and typical histories of developmental delays, intellectual/learning disabilities, and treatment-responsive anxiety/mood disorder who developed similar noteworthy neuropsychiatric expressions. We reviewed detailed neuropsychiatric histories, laboratory investigations, neuroimaging, and treatment responses and compared data for the two cases.

**Results:**

Both individuals developed treatment-resistant schizoaffective disorder in adulthood requiring multiple trials of antipsychotic treatments. While on clozapine, both patients developed parkinsonism and generalized dystonia with truncal involvement that responded to trials of low-dose levodopa without exacerbating underlying psychotic or affective symptoms.

**Conclusion:**

This report illustrates the novel occurrence of levodopa-responsive movement disorders and treatment-resistant schizoaffective disorder in individuals with WS, adding to the expanding neuropsychiatric phenotypes, and highlighting potential shared underlying mechanisms. The observed treatment response suggests that levodopa, in relatively low doses, may be safe and useful in ameliorating presumed antipsychotic-associated parkinsonism and tardive dystonia in WS.

**Supplementary Information:**

The online version contains supplementary material available at 10.1007/s10072-024-07705-3.

## Introduction

Williams syndrome (WS; OMIM #194,050), caused by submicroscopic chromosome 7q11.23 deletion, is a neurodevelopmental disorder estimated to occur in 1 in 7,500 live births [1]. While individuals with WS often have characteristic facial features, cognitive patterns, and psychosocial traits, the deletion has variable penetrance and expressivity, and WS can affect most organ systems. Thus, multidisciplinary and individualized management is often required [1].

Neurologically, many individuals with WS demonstrate features of aberrant development and maturation [1, 2]. Movement abnormalities such as developmental coordination disorder, balance difficulties, and intention tremor have been reported [3]; however, to our knowledge, other movement disorders such as dystonia and parkinsonism have not been described in WS. In terms of psychopathology, anxiety disorders are the most common followed by attention-deficit/hyperactivity disorder, depressive disorder, and obsessive-compulsive disorder. Psychotic disorders are considered rare [4, 5]. The paucity of reported neuropsychiatric manifestations in adults with WS limits our understanding of genotype-phenotype correlations and our ability to manage these manifestations effectively and safely.

Here, we describe two cases of WS who developed levodopa-responsive dystonia and parkinsonism while receiving clozapine treatment for schizoaffective disorder, suggesting expanded WS phenotypes and offering potential therapeutic options.

## Case reports

### Case 1

Case 1 was a 53-year-old female with longstanding clinical diagnosis of WS and associated *de novo* 7q11.23 deletion confirmed at age 32 years by fluorescence in-situ hybridization (FISH) assay using an elastin probe. Microcephaly and developmental delay were noted in childhood. Neurocognitive evaluation at age 28 years revealed mild to moderate intellectual impairment with relatively developed verbal abilities. Relevant aspects of the medical history are outlined in Table 1.

**Table 1:** Comparison of clinical features of two adults with Williams syndrome (WS).

Features	Case 1 (53/F)	Case 2 (49/F)
Age at clinical diagnosis of WS, in years	2	Childhood
Age of molecular confirmation of *de novo* 7q11.23 deletion, in years	32	24
Neurocognitive features	Developmental delay Mild to moderate intellectual disability	Developmental delay Mild intellectual disability Learning disabilities Dyslexia
Other multisystem manifestations	Hypertension Dyslipidemia Impaired glucose tolerance Hypothyroidism Paroxysmal atrial tachycardia Ectopic left kidney Bilateral inguinal hernia Legg-Perthes disease	Hypertension Dyslipidemia Type 2 Diabetes Mellitus (T2DM) Obesity Congenital supravalvular aortic stenosis with pulmonary artery stenosis Hemorrhoids
Family history of psychotic or movement disorders	No	No
Age at diagnosis of anxiety/mood disorder, in years	19	24
Age met criteria for schizoaffective disorder, in years	34	33
Prior antipsychotic medications	Haloperidol Chlorpromazine Thioridazine Perphenazine* Quetiapine* Olanzapine	Quetiapine* Aripiprazole Paliperidone
Age at onset of dystonia, y	48	47
Temporal evolution of dystonia	Sudden development after ~ 11 years on clozapine treatment with no identifiable changes in health status or treatment regimen	Insidious development over weeks after replacing previous antipsychotics with olanzapine, with further progression after switching to clozapine. Coincident with sudden onset of T2DM requiring metformin, linagliptin, and empagliflozin
Body distribution of dystonia	Generalized without leg involvement	Generalized with leg involvement
Psychiatric medications at dystonia presentation	Clozapine 500 mg and 525 mg on alternating days Sertraline 125 mg/day Lorazepam 1 mg thrice daily	Olanzapine 20 mg/day switched to Clozapine 400 mg/day Sertraline 200 mg/day Lorazepam 1 mg twice daily
Other movement disorders	Parkinsonism Tardive dyskinesia Hand myoclonus	Parkinsonism Tardive dyskinesia
MDS-UPDRS III score before levodopa	30	32
MDS-UPDRS III score on levodopa	22	26
FMDRS score before levodopa	13	20
FMDRS score on levodopa	9	9
Levodopa/carbidopa daily dose leading to dystonia improvement	450/112.5 mg/day	300/75 mg/day

FMDRS, Fahn-Marsden Dystonia Rating scale; MDS-UPDRS, Movement Disorder Society-sponsored Unified Parkinson’s Disease Rating Scale

*Stabilized psychotic symptoms prior to development of treatment-resistant schizoaffective disorder

Major depressive and generalized anxiety disorders were successfully managed with standard medications from age 19 years. Worsening of mild psychotic symptoms, present since late adolescence, led to a diagnosis of schizoaffective disorder meeting Diagnostic and Statistical Manual of Mental Disorders (5th ed.; DSM-V) criteria. Numerous medications were tried (Table 1) before stabilizing on a regimen consisting of perphenazine, quetiapine, sertraline, and lorazepam. However, features of orofacial and limb dyskinesia, and head and bilateral hand tremor, believed to be drug-related, developed over time. At age 37 years, refractory psychotic symptoms prompted switching antipsychotic medications to clozapine (max dose: 600 mg/day). Attempts at dose reduction led to excessive water drinking behavior that resulted in hyponatremia and seizures. Eventual optimization of clozapine was achieved with alternating doses of 500/525 mg/day.

From age 46 years, movements became progressively slow. Two years later, sudden-onset back discomfort developed followed by a severe leftward truncal tilt, which prompted hospitalization. Laboratory investigations, brain computed tomography with angiography, and magnetic resonance imaging (MRI) revealed no causative abnormalities (Figure S1). Subsequent neurologic examination revealed a mixed movement disorder consisting of orofacial dyskinesia, limb-kinetic apraxia, myoclonus, and parkinsonism. Generalized dystonia with cervical, limb, and prominent truncal involvement was also noted (Figure S2 - Video). A trial of levodopa/carbidopa (300/75 mg/day) resulted in marked improvement of parkinsonism and partial improvement of truncal tilt (Table 1). Increased neck stiffness reported at age 50 years responded to a higher levodopa dose (450/112.5 mg/day). The neuropsychiatric profile remains stable after five years without any further changes in the neuropsychiatric treatment regimen.

### Case 2

Case 2 was a 49-year-old female with a clinical diagnosis of WS in childhood and a *de novo* 7q11.23 deletion confirmed by FISH using an elastin probe at age 24 years. The pediatric presentation included developmental delay, learning disability, dyslexia, and mild intellectual disability (ID). Other relevant co-morbid conditions are outlined in Table 1.

At age 24 years, diagnoses of generalized anxiety and major depressive disorder necessitated treatment with sertraline 175 mg/day and adjunctive lorazepam. At age 30 years, affective symptoms relapsed along with brief onset of psychotic symptoms characterized by auditory hallucinations and suicidal and homicidal thoughts. At age 33 years, recurrence of persisting psychotic symptoms led to a DSM-V diagnosis of schizoaffective disorder. Symptoms resolved with addition of quetiapine (maximum dose: 400 mg/day). However, subsequent development of non-troublesome head and limb tremor, and dyskinetic orofacial and truncal movements were noted.

At age 47 years, there were prolonged hospitalizations for acute worsening of psychosis and anxiety that coincided with the onset of type 2 diabetes mellitus requiring three oral hypoglycemic agents. Severe psychotic symptoms failed to respond to quetiapine and aripiprazole. Paliperidone improved the psychosis but was discontinued due to disabling parkinsonism. After a brief trial of olanzapine, antipsychotic treatment was switched to clozapine due to poor symptom control and emergence of a slight leftward truncal tilt. While on clozapine, the truncal tilt progressed and failed to improve with lorazepam. Detailed movement disorder assessment showed mild features of parkinsonism and pronounced dystonia characterized by leftward truncal flexion (35–40°) with concomitant cervical, left upper limb, and left foot involvement (Table 1). Laboratory investigations were nondiagnostic, and brain and thoracolumbar spine MRI revealed no structural abnormalities (Figure S1). The clozapine dose was maintained given good control of psychosis. A trial of levodopa/carbidopa 300/75 mg/day provided definite, albeit partial, improvement of both parkinsonism and dystonia (Table 1). Both movement and schizoaffective disorders remain stable after two years on the regimen with notably improved functioning.

## Discussion

To our knowledge, this is the first report of levodopa-responsive dystonia, parkinsonism, and treatment-resistant schizoaffective disorder in WS, indicating novel neuropsychiatric expressions in this rare genetic disorder.

The two cases show marked similarities with only few notable differences (Table 1). Both are middle-aged women with molecularly confirmed WS who presented with mild parkinsonism and axial-predominant generalized dystonia in the context of antipsychotic therapy, with clinical data supporting diagnostic consideration of drug-induced parkinsonism and tardive dystonia. Formal criteria for tardive movement disorders require persistence of the abnormal movement(s) for at least one month after discontinuation of the offending drug(s) [6]. In both cases, however, the potential risks associated with destabilizing the severe psychotic illness far outweighed any attempt to strengthen the diagnosis of tardive dystonia by drug cessation.

The two cases followed a similar trajectory of developmental delay and ID in childhood, and diagnosis of treatment-resistant schizoaffective disorder in adulthood after longstanding well-managed mood disorders. Understanding the evolution of psychiatric diagnoses and response to treatments were aided by reliable clinical information provided by patients and caregivers, and long duration of follow-up (Table 1). These key factors, coupled with use of established diagnostic criteria, enhanced diagnostic certainty. Our cases add to earlier reports of psychotic illnesses in WS with diagnoses ranging from depression with psychotic features to schizophrenia [4, 5]. Collectively, these cases highlight the value of longitudinal assessment and formal diagnostic criteria to distinguish illnesses requiring long-term treatment from short-lived symptoms, and to appreciate antipsychotic treatment effects, particularly for a rare and complex genetic disorder.

Longitudinal follow-up also facilitated appreciation of the temporal evolution of movement disorders, and the similarities and differences between the cases. Intriguingly, movement disorders developed or worsened on clozapine, a drug known to exhibit the least neuromotor side effects [7]. One plausible explanation is that the biochemical lesion leading to the movement disorders stemmed from previous exposure to other antipsychotics rather than, or in addition to, clozapine. Larger studies are needed to dissect factors that modify the predisposition and clinical course of movement disorders in WS in the presence and absence of antipsychotic exposure.

While movement disorders, treatment-resistant schizoaffective disorder, and WS could each represent unrelated disease processes, one can speculate about potential shared underlying pathophysiological mechanisms. Neurochemical imbalances in acetylcholine and dopamine are implicated in tardive dystonia [8], and may also play a role in treatment-resistant psychosis and WS. Reduced striatal cholinergic neuron density was demonstrated in post-mortem examinations of 13 adult WS cases [9] and have been similarly observed in schizophrenia [10] and tardive movement disorders [11]. Intuitively, this reduction may lead to altered central nervous system (CNS) cholinergic neurotransmission in WS and set the stage for tardive dystonia development with antipsychotic treatment. This possibility deserves further exploration. Dopamine dysregulation plays a key role in psychotic illnesses and recent data for schizophrenia suggest a link to aberrant neurodevelopment and synaptic homeostasis [12]. Notably, abnormal neurodevelopment occurs in WS possibly arising from reduced dosage of genes within the deletion region, including *GTF2I*,* GTF2IRD1*,* LIMK1*, and *CLIP2* [1, 2]. This could conceivably play a role in predisposition to parkinsonism and tardive dystonia in the setting of antipsychotic treatment. Further studies are needed to assess for dopamine dysregulation in WS and whether gene dosage reduction at 7q11.23 plays a role in late-occurring neuropsychiatric phenotypes.

Aging and chronic physical illness are also known to increase the risk of developing movement disorders by weakening defense systems against antipsychotic-related free radical formation and excitotoxicity [11]. A recent study involving 32 patients showed evidence of accelerated biological aging in WS [13], and this mechanism combined with the associated multisystemic illnesses may have increased the propensity for antipsychotic-related movement disorders in both of our cases. It also remains possible that movement disorders developed due to the combined effects of multiple microstructural, neurochemical, and functional perturbations.

The mechanism behind the observed positive response of the movement disorders to levodopa is of interest, particularly as there is yet no clear role for levodopa therapy in drug-induced and tardive syndromes [14, 15]. It is tempting to speculate that low-dose levodopa may have restored normal dopamine receptor sensitivity and cholinergic-dopaminergic equilibrium in the CNS. This possibility warrants further investigation.

Our report illustrates the novel occurrence of levodopa-responsive dystonia and parkinsonism in two adults with WS managed for treatment-resistant schizoaffective disorder, adding to the expanding phenotypes of WS and suggesting potential shared underlying mechanisms for late-occurring neuropsychiatric manifestations. Importantly, our experience suggests that levodopa, in relatively low doses, may be useful in ameliorating antipsychotic-associated movement disorders without exacerbating psychotic or affective symptoms in WS. As for other multi-gene genomic disorders, follow-up into adulthood can reveal a lifelong trajectory of associated features and the value of standard management for each [16].

## Electronic supplementary material

Below is the link to the electronic supplementary material.

Supplementary Material 1

Supplementary Material 2: On examination of Case 1 at age 49 years (prior to levodopa therapy), there was a mild truncal postural tilt to the left (~ 20°) with diminished swing of both arms, as well as tonic left elbow and bilateral wrist flexion during ambulation. While seated and with arms outstretched, there were fast, arrhythmic, jerks involving the bilateral distal upper extremity representing myoclonus. In position of repose, there was also evidence of anterocollis, left laterocollis, and a slight rightward head tilt. There was mild limb-kinetic apraxia on the left. Examination before initiation of levodopa showed parkinsonism characterized by limb rigidity, left > right limb bradykinesia, and postural instability on retropulsion

## Data Availability

Relevant clinical, genetic, and imaging data are presented in the text and supplementary material. Further inquiries can be directed to the corresponding author.
