# Supplementary material for: Levodopa-responsive dystonia, parkinsonism, and treatment-resistant schizoaffective disorder in Williams syndrome
Source: Neurol Sci. 2024 Jul 18;46(1):463–8. doi: 10.1007/s10072-024-07705-3 (PMC11698793; doi:10.1007/s10072-024-07705-3)
Supplement: Supplementary file 1 — Supplementary Material 1 [file 10072_2024_7705_MOESM1_ESM.docx]

**Supplementary Figures**

**
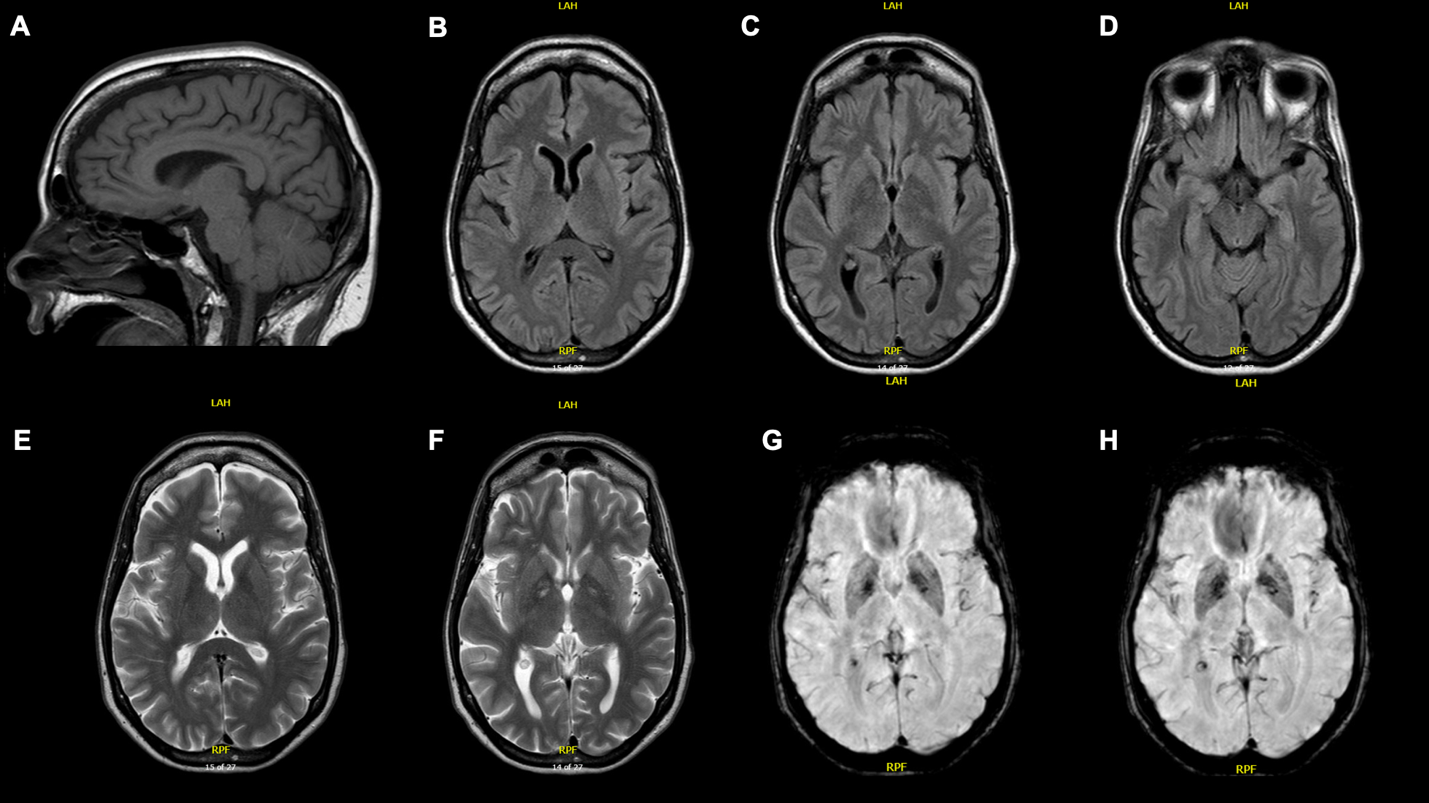
**

**Figure S1.** Cranial magnetic resonance imaging for Case 1, which did not show structural lesions to explain dystonia and other co-occurring movement disorders (A: sagittal T1-weighted sequence; B-D: axial T2 fluid inversion recovery sequences; E-F: axial T2-weighted sequences; G-H: axial gradient recall echo sequences).

**
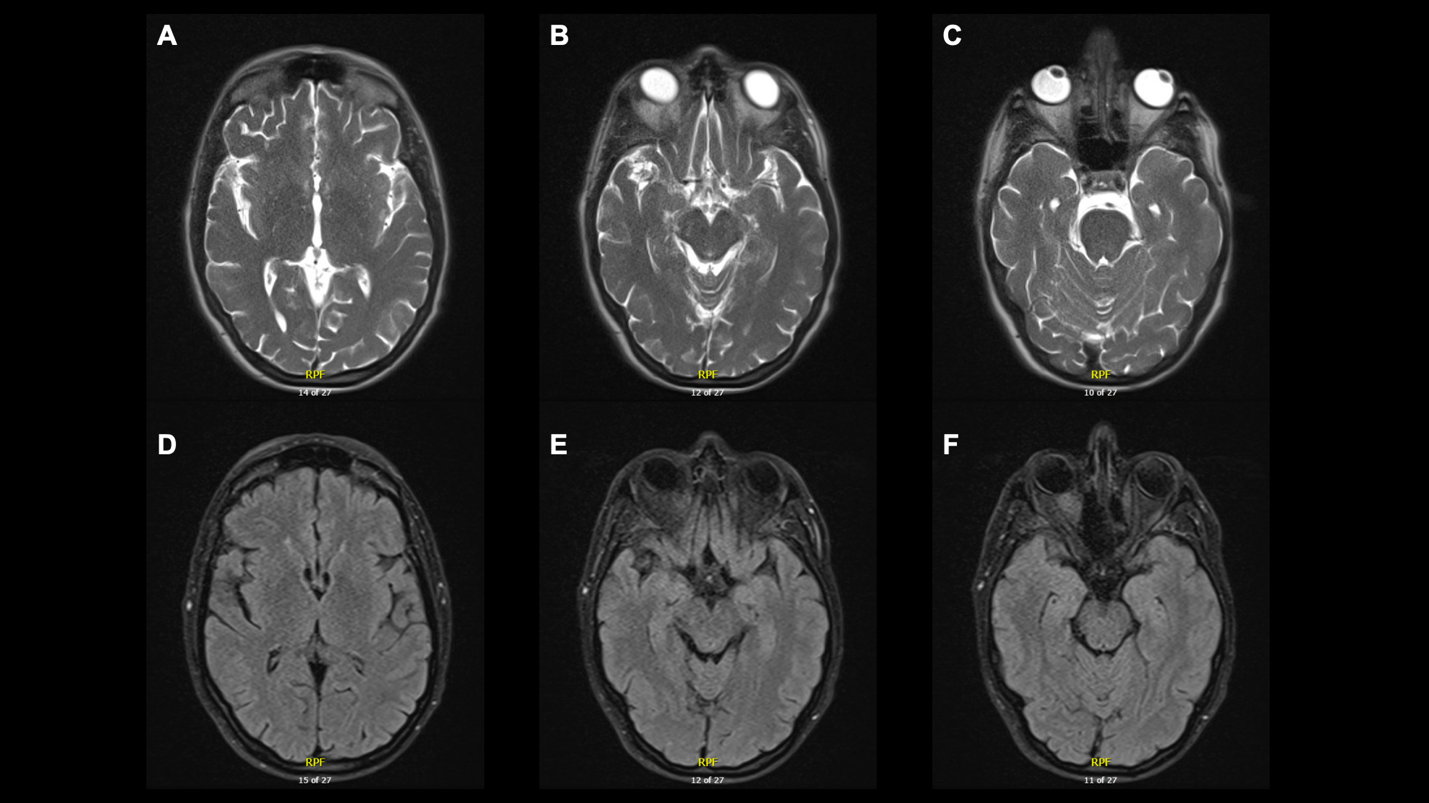
**

**Figure S2.** Cranial magnetic resonance imaging for Case 2, which did not show structural lesions to explain dystonia and other co-occurring movement disorders (A-C: axial T2-weighted sequences; D-F: axial T2 fluid inversion recovery sequences).
